# Heavy Metal Concentration in Swiss Chard (*Beta vulgaris* ssp. *cicla* L.) Cultivated Along River Banks in Addis Ababa, Ethiopia

**DOI:** 10.1155/ianc/1246637

**Published:** 2026-06-28

**Authors:** Betelhem Tefera, Merid Tessema, Mesfin Hailemariam Habetgebriel

**Affiliations:** ^1^ Department of Chemistry, Addis Ababa University, Addis Ababa, Ethiopia, aau.edu.et; ^2^ Geochemical Research Laboratory, Geological Institute of Ethiopia, Addis Ababa, Ethiopia; ^3^ Department of Microbial Sciences and Genetics, Addis Ababa University, Addis Ababa, Ethiopia, aau.edu.et; ^4^ Crops Breeding and Genetics, Ethiopian Institutes of Agricultural Research, Addis Ababa, Ethiopia

**Keywords:** digestion, optimization, selected metals, spectrophotometry, Swiss chard

## Abstract

This study employed flame atomic absorption spectrophotometry to determine the levels of selected heavy metals in Swiss chard. Samples were collected from three vicinities of Addis Ababa: Akaki, Sebeta, and Kotebe. A 0.5‐g dried and powdered sample was analyzed using the wet digestion method with 69%–72% HNO_3_ and 70% HClO_4_, with optimized digestion. The calibration curves and coefficient (*r*) value were between 0.996 and 0.999, showing very good linearity. The accuracy of the optimized procedure was tested using samples that had a known amount of the substance added. The recovery percentages ranged from 95.89% to 100%, which is a good range. The mean concentrations (mg/kg) of nickel (0.017) and zinc (0.088) in the Swiss chard were determined . The mean concentrations of metals in Swiss chard from the three areas indicated a higher concentration of zinc in Kotebe compared to Akaki and Sebeta. A higher concentration of nickel (Ni) was found in Akaki’s Swiss chard compared to Sebeta and Kotebe. The Pearson correlation coefficients of metals from the Swiss chard between nickel and zinc showed a very strong correlation. The best approach combines immediate risk reduction such as cleaner irrigation and consumer warnings, with long‐term remediation such as soil treatment, pollution control, and policy enforcement to protect both farmers and consumers.

## 1. Introduction

Heavy metals and metalloids are natural, dense elements (> 5 g/cm^3^) that are non biodegradable and persistent in the environment. Consequently, remediation strategies are essential to prevent their leaching and mobilization into other environmental components [1]. Heavy metals are frequently reported as potential hazards in contaminated vegetables especially in industrial areas [2, 3]. On the contrary, Ullah et al. [4] directly identified these dangerous heavy metal ions at low concentrations in various samples. Areas with potential hazards and occurrences in contaminated vegetables include cadmium, zinc, lead, nickel, and chromium [5]. Heavy metals can be entered into the human body through inhalation of dust, direct ingestion of soil, and consumption of food plants grown in metal‐contaminated soil, thereby causing diseases [6]. Fruits and vegetables are the primary pathways of heavy metal exposure to humans via ingestion [7]. Fresh fruits and vegetables are important as they contain vitamins, minerals, and other components that promote health and the prevention of various diseases [8]. The concentration of heavy metals in plants is perhaps influenced by the nature of the soil, environmental pollution, and the time of harvesting [9]. Excessive phosphate fertilizer usage may increase metal concentration in agricultural soils [10]. The use of fertilizers could also contribute to metal pollution in vegetables [11]. Heavy metal buildup in plants can lead to serious problems for both the environment and people’s health over time.

Metals can be transferred from soil into plants through the roots in the form of dissolved ions via a series of complex processes [12]. The accumulation of contaminants in plants can be estimated by the metal mobilization from soil to plant [13–16]. Previous studies have discovered high metal enrichment in leafy vegetables, followed by tubers and fruits [17–19]. Agrawal et al. [20] found that cadmium and lead concentrations are higher in leafy vegetables than in tubers. They are made up chiefly of cellulose, hemicellulose, and pectin substances that give them their texture and firmness [7].

Heavy metals are given significant interest throughout the globe due to their toxic, mutagenic, and teratogenic effects even at very low concentrations (Dasharathy et al. [21]). Eating fruits and vegetables that have been polluted with heavy metals is the most common way people get exposed to these harmful substances [22]. The conventional techniques employed for the elimination of heavy metals are deemed inadequate when the heavy metal concentration is less than 100 mg/L [23]. The chemical composition of vegetables shows high water content, sugars, protein, starch, fat, and energy value [10].

High concentrations of heavy metals in fruits and vegetables such as cadmium and lead have been shown to have carcinogenic effects and are related to the high prevalence of upper gastrointestinal cancer [18, 24]. The primary objective of this research is to determine the presence of heavy metals in Swiss chard vegetables collected from the Akaki, Sebeta, and Kotebe areas in and near Addis Ababa.

## 2. Methodology

### 2.1. Descriptions of the Study Area

**FIGURE 1:**
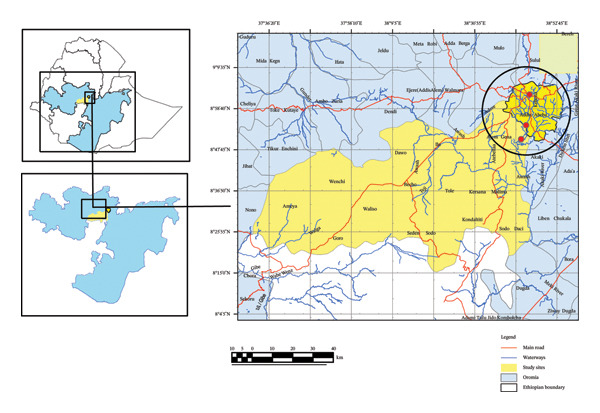
Sampling locations for Swiss chard along three river banks near Addis Ababa, Ethiopia.

This study was executed in Addis Ababa University, Department of Chemistry, Analytical Chemistry Research Laboratory, and samples of Swiss chard were obtained from three separate areas: Akaki (9°12′52″N, 38°59′38″E), Sebeta (8°54′40″N, 38°37′17″E), and the Kotebe river (8°58′0″N, 38°49′60″E), as shown in Figure 1. These sites sell a variety of vegetable goods, including Swiss chard, to the market. The sampling method was chosen based on Swiss chard production and the risks of heavy metal contamination.

### 2.2. Sample Collection Sites and Preparation

Two kilograms of Swiss chard leaf samples were collected from the Akaki, Sebeta, and Kotebe markets, packed, labeled, and transported to the Analytical Chemistry Laboratory of Addis Ababa University (see Supporting Figure S1). The edible components of the samples were then cleaned with deionized water, while nonedible parts were removed. The washed Swiss chard veggies were chopped into small pieces with a knife to allow for air drying and placed in an electric oven at 105°C for 2 days. The desiccated samples were subjected to manual grinding/milling with a mortar and pestle into a fine‐textured powder, which was then passed through a 2‐mm sieve. The resulting powder was stored at room temperature in an air‐tight polythene pouch before being transported to the laboratory for digestion and metal analysis.

### 2.3. Chemicals and Reagents

The Swiss chard was digested using nitric acid (HNO_3_) (69%–72% concentration) and 70% perchloric acid (HClO_4_). Stock standard solutions of 1000 mg/L, including 2% HNO_3_, of the metals Pb, Zn, Cd, Cr, and Ni were made just before analysis for calibration and spiking of the experiments.

### 2.4. Instrument and Apparatus

The Kjeldahl apparatus (Gallenkamp, England) was used to digest the materials. We used an oven (J.P. Selecta, S.A., Spain) to dry the samples, a ceramic mortar and pestle to grind the samples to the required fine powder size, a digital analytical balance (Mettler Toledo, Model At250, Switzerland) to weigh the samples accurately, and filter paper (Whatman No. 42, Cytiva, United Kingdom) to separate the filtrate from the residue. Heavy metal concentrations were measured using a technique called flame atomic absorption spectrophotometry with the ZEEnit 700P instrument (Analytik Jena, Germany) (see Supporting Figure S2).

### 2.5. Optimization of Digestion Procedure

To get a clear sample that works well with flame atomic absorption spectrophotometry, different ways of digesting Swiss chard samples were tested. These methods used a mix of concentrated 69% nitric acid and 70% perchloric acid. The amount of acid mix, the temperature, and the time were changed to find the best way. The best results came from using 4 mL of nitric acid and 1 mL of perchloric acid, with a digestion temperature of 300°C for 3 h (see Supporting Tables 1–3).

### 2.6. Digestion of the Swiss Chard

The optimized conditions for 0.5 g of dried, powdered, and homogenized Swiss chard were weighed and transferred to a 250‐mL round‐bottomed flask. The samples were mixed with 4 mL of HNO_3_ and 1 mL of HClO_4_ in a 4:1 volume ratio. The solutions were processed using Kjeldahl digestion equipment with a reflux condenser at a temperature of 300°C for 3 h. After the digestion was finished, a clear and colorless solution was produced. The solution was then allowed to cool for 15 min before the condenser was removed from the flask, followed by an additional 5 min. After adding 5 mL deionized water to the sample’s cooled solution to dissolve any precipitate that had developed during cooling, the mixture was gently swirled and filtered into a 50‐mL volumetric flask using Whatman filter paper (No. 42) to remove any suspended or turbid materials. Finally, the clear solution was diluted to the 50 mL mark of the volumetric flask with deionized water. To ensure the precision of the results, each Swiss chard vegetable sample was digested and analyzed in triplicate. The blank solution was created by using the same number of reagents in all procedures. Flame atomic absorption spectroscopy (FAAS) was used to assess the concentration of heavy metals in the filtrate.

### 2.7. Standards for Sample Preparation and Analysis

The development of affordable, eco‐friendly sample preparation method with analytical figure of merit is of great interest to analytical chemists [4]. The 100‐mg/L metal stock solutions were diluted with deionized water to form a series of working standard solutions of each heavy metal element in a sample for flame atomic absorption spectrophotometry. Calibration curves were created for each element independently, using standards of known concentration. For Cr, Zn, Cd, Ni, and Pb, calibration curves were generated at four concentration levels: 0.25, 0.50, 0.75, and 1.00 mg/L. Blank readings were also taken after the correction was applied during the calculation of the concentrations of various elements.

#### 2.7.1. Reagent Blanks

A reagent blank is a solution that has all of the same components as the sample solution but no identifiable analyte materials. The reagent blank recognizes the quantity of signal produced by the reagents used to prepare the samples. In this investigation, a reagent blank was created for each set of measurements.

#### 2.7.2. Flame Atomic Absorption Spectrophotometry

Heavy metal identification in Swiss chard was performed using flame atomic absorption spectrophotometry via a wet optimized digestion procedure. The measurements of Zn, Ni, Cd, Cr, and Pb elements were made using a conventional cathode lamp, and all of the samples were examined using flame atomic absorption. The flame atomic absorption spectrophotometry under operating circumstances is displayed in Table 1.

**TABLE 1:** Instrumental operating conditions for the determination of heavy metals by flame atomic absorption spectroscopy (FAAS).

Metals	Wavelength (nm)	Slit width (nm)	Lamp current (mA)	Flame type	Support
Zink	213.9	1.0	5	Air‐acetylene	Nitrous oxide
Cadmium	283.2	0.5	5	Air‐acetylene	Nitrous oxide
Chromium	228.8	0.5	4	Air‐acetylene	Nitrous oxide
Nickel	357.9	0.2	7	Air‐acetylene	Nitrous oxide
Lead	232.0	0.2	4	Air‐acetylene	Nitrous oxide

Abbreviations: mA = milliampere, nm = nanometer.

#### 2.7.3. Method Validation

Method validation results can help to evaluate the quality, consistency, and reliability of analytical outcomes, which is an essential component of any effective analytical practice. The analytical method was validated by assessing the following parameters: limit of detection (LOD), limit of quantification (LOQ), precision, and accuracy.

#### 2.7.4. LOD and LOQ

The LOD is the smallest amount of a substance that can be told apart from random changes in a test result from a blank sample. It is usually based on the level found in the blank solution at a certain time. The calculation is done by multiplying the pooled standard deviation of the reagent blank (SD blank) by three, which is written as LOD equals three times SD blank. The standard deviation of the calibration blank is shown as (SD blank), as mentioned in [26]. The LOQ means the smallest amount of a substance that can be measured with enough accuracy and precision to be considered reliable. The LOQ is found by multiplying the standard deviation of the blank solution by 10, where SD is the standard deviation of the blank, as shown in Table 2.

**TABLE 2:** Limits of detection and quantification of heavy metals in Swiss chard.

Metal	Standard deviation of the blank	The limit of detection (mg/kg)	The limit of quantification (mg/kg)
Zinc (Zn)	0.014	0.042	0.140
Nickel (Ni)	0.006	0.018	0.060

Abbreviation: mg/kg = milligrams per kilogram.

### 2.8. Statistical Analysis

The experimental data were subjected to one way analysis of variance (ANOVA), and if the results showed significant differences (*p* < 0.05), the correlation coefficient of relations with heavy metals was evaluated using Pearson correlation coefficient methods, and the graphical representation of data was done by Microsoft Excel 7. The results for heavy metals were given as mean values with their respective standard deviation. For all tests, statistical significance was considered at the *p* < 0.05 level.

## 3. Results and Discussion

### 3.1. Instrument Calibration

The working standards for each metal were prepared by serial dilution with deionized water from a 1000‐mg/L standard stock solution, and each was placed individually in the FAAS. The linear correlation coefficient (*r* ^2^) of the standard curves varied from 0.996 to 0.099, indicating a strong linearity. Calibration yielded a minimum correlation coefficient (*R* ^2^) of 0.995. Table 3 lists the concentrations and correlation coefficients of the calibration standards used.

**TABLE 3:** Calibration standards’ concentrations and correlation coefficients.

Elements	Wavelength (nm)	Concentration of working standards (mg/L)	Regression equations (*Y* = *m* *x* + *b*)^∗^	*R* ^2^
Zinc	213.9	0.25, 0.5, 0.75, 1	*Y* = 0.2529*x* + 0.0339	0.9999
Nickel	357.9	0.25, 0.5, 0.75, 1	*Y* = 0.1064*x* + 0.0018	0.9937
Cadmium	283.2	0.25, 0.5, 0.75, 1	*Y* = 0.1924*x* + 0.0103	0.9978
Chromium	228.8	0.25, 0.5, 0.75, 1	*Y* = 0.1108*x* + 0.0024	0.9995
Lead	232.0	0.25, 0.5, 0.75, 1	*Y* = 0.0261*x* − 0.0020	0.9926

*Note: m* = slope; *x* = concentration (mg/L); *b* = intercept.

Abbreviations: mg/L = milligrams per liter, nm = nanometer.

^∗^ *Y* = intensity.

### 3.2. Comparison of Metal Concentrations in Swiss Chard From the Different Areas

**FIGURE 2:**
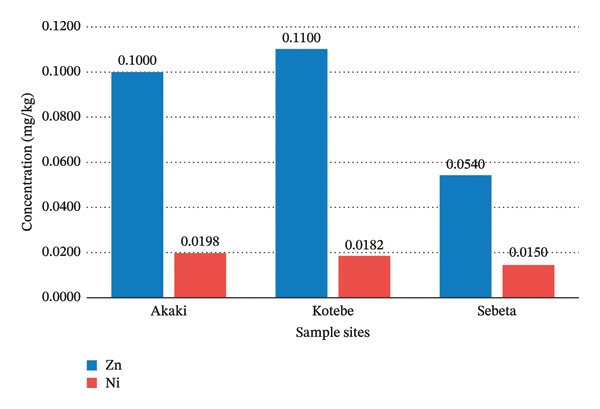
Evaluation of Zn and Ni in Swiss chard from three locations of Akaki, Sebeta, and Kotebe.

According to Table 4 and Figure 2, the metal concentrations in Swiss chard from different locales were compared. From Table 4, the flame atomic absorption spectrophotometry only detects the elements such as Zn and Ni; the remaining three tested elements could not be detected.

**TABLE 4:** Mean concentrations of metals in Swiss chard vegetable.

Element	Sebeta		Akaki		Kotebe	
	Mean ± SD	RSD (%)	Mean ± SD	RSD (%)	Mean ± SD	RSD (%)
Zinc	0.054 ± 0.004	7.89	0.10 ± 0.0054	5.36	0.11 ± 0.0014	1.27
Nickel	0.015 ± 0.000085	0.56	0.0198 ± 0.00075	3.78	0.018 ± 0.00037	2.04
Cadmium	ND	ND	ND	ND	ND	ND
Chromium	ND	ND	ND	ND	ND	ND
Lead	ND	ND	ND	ND	ND	ND

Abbreviations: ND = not detected, RSD = relative standard deviation, SD = standard deviation.

As demonstrated in Figure 2, the nickel concentration in Akaki Swiss chard vegetable is higher than that of Sebeta and Kotebe Swiss chard vegetables. The average amount of nickel in Akaki is more than in Sebeta Swiss chard, and the amount in Sebeta is more than in Kotebe Swiss chard. The average nickel values in Swiss chard from Sebeta, Akaki, and Kotebe were 0.015, 0.0198, and 0.018293 mg/kg, respectively. This result is below the limit or it is the limit. As demonstrated in Figure 2, the Zn concentration in Kotebe Swiss chard vegetables is higher than that of Akaki and Sebeta Swiss chard vegetables. Furthermore, the average Zn concentration in Kotebe is higher than in the Akaki Swiss chard vegetable metal concentration, which is likewise higher than in the Sebeta Swiss chard vegetable sample. The average Zn concentrations in Swiss chard from Sebeta, Akaki, and Kotebe were 0.054, 0.10, and 0.11, respectively. As shown in Figure 2, the Swiss chard from Akaki and Kotebe contains a higher concentration of Ni than that from Sebeta. The Swiss chard from Kotebe has a higher concentration of Zn than that from Sebeta and Akaki. Similarly, the Swiss chard from Sebeta contains a relatively small amount of Zn compared to the Swiss chard from Akaki and Kotebe. Nickel has a relatively lower concentration in Sebeta Swiss chard compared to that from Akaki and Kotebe. The concentration of Zn decreases in the trend of Kotebe > Akaki > Sebeta. Additionally, the concentration of Ni decreases in the trend of Akaki > Kotebe > Sebeta. The mean concentration order of metal content in the Swiss chard from Sebeta is Zn > Ni, in Akaki is Zn > Ni, and in Kotebe is Zn > Ni.

### 3.3. Comparison of Heavy Metal Concentrations With Previously Reported Values

Different countries have reported the presence of selected metals in various parts of Swiss chard. As shown in Table 5, the average levels of metals found in this study using the flame atomic absorption spectrophotometry method, based on Swiss chard samples from Sebeta, Akaki, and Kotebe, are compared with values from previous studies. The level of zinc found in this study is 0.008 mg per kg in the areas of Sebeta, Akaki, and Kotebe, which is lower than what has been reported in other places, except for Zambia. In this study, the amount of Ni found was 0.017 mg per kg, which is less than what was found in most other studies, except in Zambia, Kenya, and Turkey. In this study, the levels of lead, chromium, and cadmium are all below the minimum amount that can be detected, which is similar to what has been reported in other studies. This study could not show that the levels of metals such as Pb, Mn, and Fe found in most of the vegetables examined are much higher than the maximum allowed limits set by WHO/FAO guidelines [25].

**TABLE 5:** Comparison of mean metal concentrations in Swiss chard with the reported literature.

Country	Metal concentration (mg/kg)					Method	Reference
	Zink	Nickel	Cadmium	Chromium	Lead		
Ethiopia	0.088	0.017	ND	ND	ND	FAAS	This study
Ethiopia	27.64	9.99	0.38	1.2	0.32	FAAS	Abrham and Gholap [27]
Zambia	NR	ND	NR	0.29	0.105	FAAS	Evaristo [28]
Kenya	66.21	NR	NR	NR	0.36	FAAS	Mutune et al. [29]
Turkey	2.91	NR	NR	0.007	0.106	GFAAS	Neriman [30]
Ethiopia	48.65	51.85	1.38	11.36	9.52	FAAS	Banchamlak et al. [31]

Abbreviations: FAAS = flame atomic absorption spectroscopy, GFAAS = graphite furnace atomic absorption spectrometry, ND = not detected, NR = not reported.

### 3.4. Metal Concentrations in Swiss Chard Compared to Other Vegetables Documented in Ethiopian Literature

In this study, the concentration of selected metals (Zn, Ni, Cd, Cr, and Pb) in Swiss chard leaves was determined. The comparison is based on heavy metal concentration according to the available data, which shows in Table 6 that the concentrations of Zn, Ni, Cd, Cr, and Pb are comparable with those of various other vegetables listed in the table. The concentrations of zinc and nickel were lower in Swiss chard from all studied sites than in other vegetables. The levels of Cd, Cr, and Pb in this study were all below the detection limit.

**TABLE 6:** A comparison of the metal content in Swiss chard with other vegetables was done using the flame atomic absorption spectroscopy (FAAS) method, as reported in studies from Ethiopia.

Sample type	Metal concentration (mg/kg)					Reference
	Zinc	Nickel	Cadmium	Chromium	Lead	
Sebeta Swiss chard (SSC)	0.054	0.015	ND	ND	ND	Own result
ASC	0.10	0.019	ND	ND	ND	Own result
Akaki Swiss chard, Kotebe Swiss chard	0.11	0.018	ND	ND	ND	Own result
Cabbage	NR	NR	ND	ND	ND	Getachew et al. [32]
Lettuce	23.21	15.06	0.21	1.61	0.24	[][][33]
Spinach	NR	NR	0.37	2.23	0.06	Getachew et al. [32]
Green pepper	10.17	19.82	0.20	1.73	0.24	Bagdatlioglu et al. [34]

Abbreviations: mg/kg = milligrams per kilogram, ND = not detected, NR = not reported.

### 3.5. ANOVA

In this study, one‐way ANOVA was used to examine the mean metal values of each section of the Swiss chard samples from the three sampling sites. The findings were used to demonstrate no significant variations in the mean concentrations of each metal between the studied Swiss chards. As shown in Table 7, the statistical analysis of ANOVA revealed no significant difference (*p* > 0.05) in the mean concentrations of metals such as nickel and zinc observed in Swiss chard samples from three distinct areas. Similar results were reported by Dube and Kanido [3], indicating that there is no statistically significant difference between mean values of Na, Co, Cu, Zn, TDS, pH, EC, NO_3_^−^, Cl^−^, and F^−^ of spring and well‐water samples. On the other hand, similar researchers reported significant differences (*p* > 0.05) of the average levels of K, Ca, Mg, and Fe minerals and DO, TSS, turbidity, and SO_4_^2−^ [3].

**TABLE 7:** Analysis of variance (ANOVA) of Swiss chard.

Heavy metals	*F* calculated	*F* critical	*p* value
Zinc	0.04	5.14	0.96^ns^
Nickel	0.06	5.14	0.94^ns^

*Note:* For both heavy metals, the calculated *F*‐values are much smaller than the critical *F*‐value (5.14), and the *p* values are very high (close to 1.0). This means there is no statistically significant difference between the groups being tested for either zinc or nickel.

Abbreviation: ns = not significant.

### 3.6. Pearson Correlation Coefficient for Heavy Metals

The correlation coefficient in experimental analysis shows how close the data points are to the best‐fit line, and the results obtained are presented in Table 8. The correlation results are divided into categories based on the strength of the relationships: no correlation (*r* = 0.00–0.19), low correlation (*r* = 0.20–0.39), medium correlation (*r* = 0.40–0.59), higher correlation (*r* = 0.60–0.79), and maximum correlation (*r* = 0.80–1.00) [35, 36]. Table 8 shows the Pearson correlation coefficient between the metal levels found in the Swiss chard samples used in this study. The strongest link was between nickel and zinc, with a correlation of 0.84. In this study, the levels of lead, chromium, and cadmium were all lower than what can be measured. In their work, Lemessa et al. [2] reported that the heavy metals such as Cr and Cd concentrations with Pb concentrations had shown a strong positive association in vegetables during May in Bole Lemi Industrial Park in Addis Ababa.

**TABLE 8:** Correlation coefficients of the five heavy metal pollutants in Swiss chard.

Metals	Zn	Ni	Cd	Cr	Pb
Zn	1				
Ni	0.84	1			
Cd	ND	ND	1		
Cr	ND	ND	ND	1	
Pb	ND	ND	ND	ND	1

Abbreviation: ND = not detected.

### 3.7. Limitations of the Study

The study has some limitations that have to be improved. The number of samples tested is only five, which is very small. Also, the instrument used to test the heavy metals, which is an atomic absorption spectrometer (AAS), could not detect some elements such as lead and chromium. On the other hand, it could detect some heavy metals like mercury (Hg), arsenic (As), and iron (Fe). Also, the sampling areas were not wide or representative. In addition, this study only looked at the leaf part of the Swiss chard.

## 4. Conclusion

This experiment confirmed the levels of selected heavy metals in Swiss chard samples collected from three districts within Addis Ababa city administration and its surrounding areas. Using FAAS, an analysis was performed on the Swiss chard to determine its levels of heavy metals. However, the elements cadmium (Cd), chromium (Cr), and lead (Pb) were not found. The digestion approach for detecting metals in Swiss chard was optimized and confirmed using a spiking method, resulting in high percentage recoveries of 95.8% for nickel and 100% for zinc. The average metal concentration (mg/kg) in Swiss chard sampled from Sebeta, Akaki, and Kotebe was zinc > nickel. The concentration of zinc declined from Kotebe to Akaki to Sebeta, while nickel decreased from Akaki to Kotebe to Sebeta. Nickel (Ni) and zinc (Zn) exhibited the highest correlation values among the metals from the three sites, as indicated by the Pearson correlation coefficients.

### 4.1. Future Research Directions

Researchers undertaking similar work should focus on enlarging the sample size for AAS testing, which should include heavy metals such as mercury (Hg), arsenic (As), and iron (Fe), while ensuring that sampling areas are broadly selected across urban, semiurban, and industrial sites. For the AAS‐undetected elements such as lead and chromium, the high‐throughput instruments such as inductively coupled plasma mass spectrometry (ICP‐MS) or graphite furnace atomic absorption spectrometer (GFAAS) should be used. In addition, this study only uses leaf part of the Swiss chard; therefore, future research should also need to include stems, roots, and even soil samples from the study area to provide better results and information.

## Funding

The authors declare that this article’s research did not receive any financial support.

## Disclosure

This work is the first author’s thesis [37].

## Conflicts of Interest

The authors declare no conflicts of interest.

## Supporting Information

Additional supporting information can be found online in the Supporting Information section.

## Supporting information


**Supporting Information 1** Supporting Figure S1. The Swiss chard sample for the three river banks in the vicinity of Addis Ababa. Supporting Figure S2. Atomic absorption spectrophotometry that was used for the experiment.

**Supporting Information 2** Supporting Table 1. Optimization of the volume ratio of the reagents for a 0.5‐g sample digestion. Supporting Table 2. Optimization of the digestion temperature for a 0.5‐g sample. Supporting Table 3. Optimization of the digestion time.

## Data Availability

The authors will provide the raw data supporting this article’s conclusions without undue reservation.
