# Supplementary material for: Heavy Metal Concentration in Swiss Chard (Beta vulgaris ssp. cicla L.) Cultivated Along River Banks in Addis Ababa, Ethiopia
Source: Int J Anal Chem. 2026 Jun 28;2026:1246637. doi: 10.1155/ianc/1246637 (PMC13310366; doi:10.1155/ianc/1246637)
Supplement: Supplementary file 1 — Supporting Information 1 Supporting Figure S1. The Swiss chard sample for the three river banks in the vicinity of Addis Ababa. Supporting Figure S2. Atomic absorption spectrophotometry that was used for the experiment. [file IANC-2026-1246637-s001.pdf]

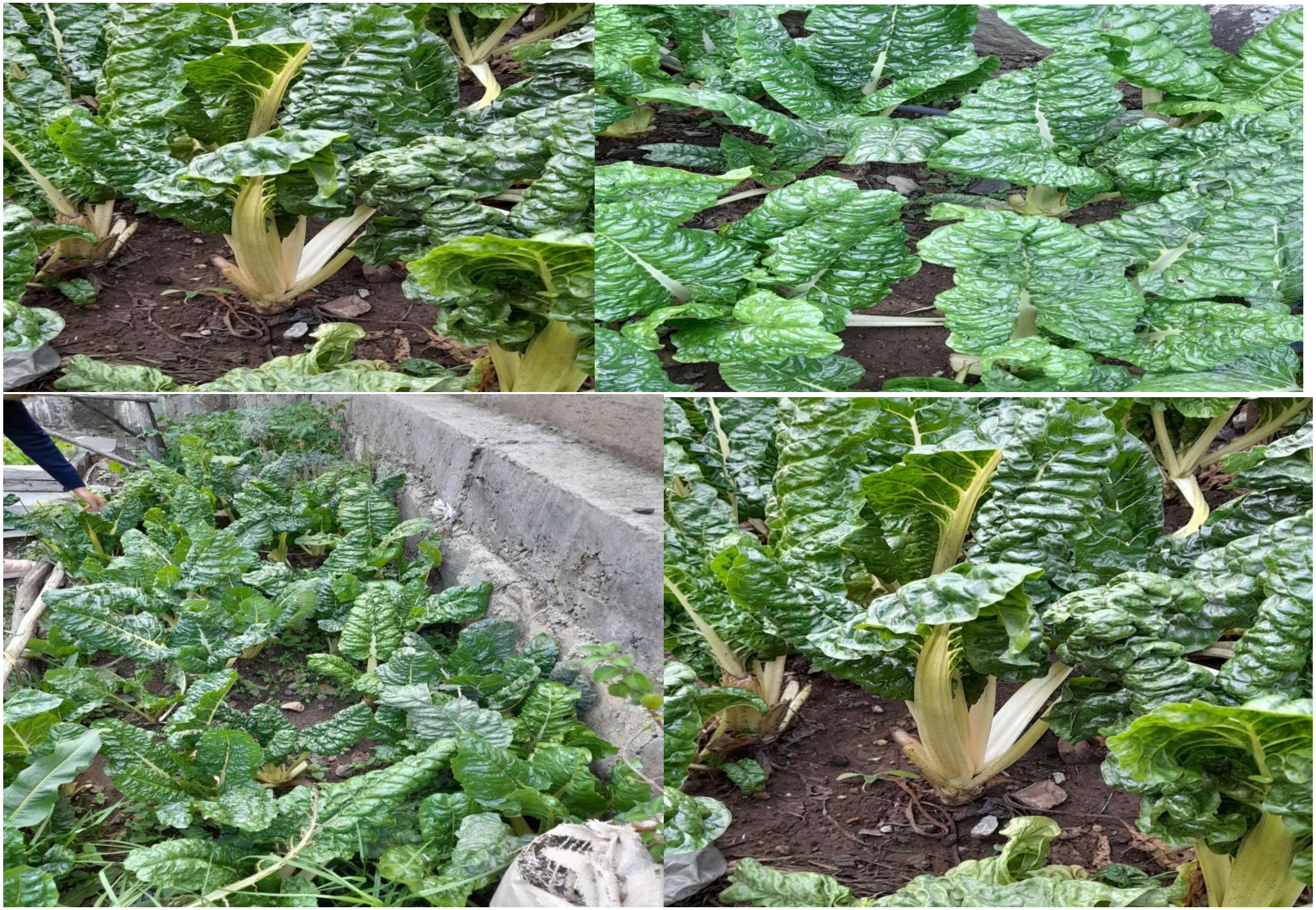

SUPPLEMENTARY FIGURE S1. The Swiss chard sample for the three river banks in the vicinity of Addis Ababa. Photo credit: Betelhem Tefera

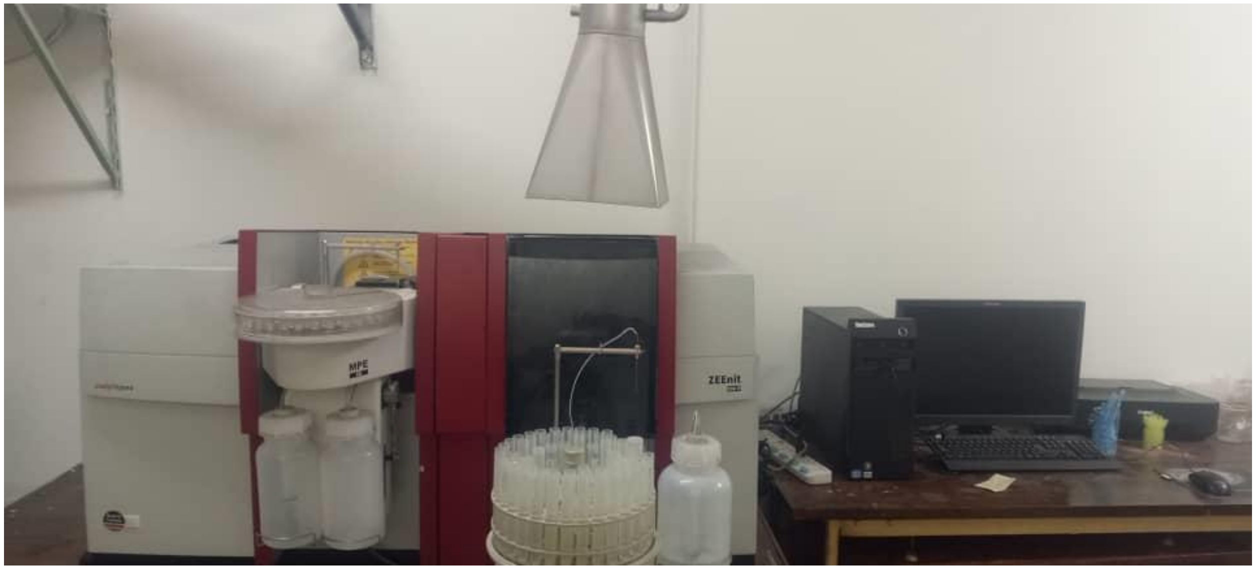

SUPPLEMENTARY FIGURE S2. Atomic Absorption Spectrophotometry that used for the experiment.

(Photo credit: Betelhem Tefera)
