# Supplementary material for: Heavy Metal Concentration in Swiss Chard (Beta vulgaris ssp. cicla L.) Cultivated Along River Banks in Addis Ababa, Ethiopia
Source: Int J Anal Chem. 2026 Jun 28;2026:1246637. doi: 10.1155/ianc/1246637 (PMC13310366; doi:10.1155/ianc/1246637)
Supplement: Supplementary file 2 — Supporting Information 2 Supporting Table 1. Optimization of the volume ratio of the reagents for a 0.5‐g sample digestion. Supporting Table 2. Optimization of the digestion temperature for a 0.5‐g sample. Supporting Table 3. Optimization of the digestion time. [file IANC-2026-1246637-s002.pdf]

SUPPLEMENTARY TABLE 1. Optimization of the volume ratio of the reagents for a 0.5 g sample of digestion

№	Total volume	Volume ratio in ml HNO3: HClO4	Time	Temperature (˚C)	Observation during the volume ratio in optimization
1	3	2:1	3:00	300	Deep yellow solution
2	3	1.5:1.5	3:00	300	Deep yellow solution
3	4	3:1	3:00	300	Light yellow solution
4	4	2.5:1.5	3:00	300	Yellow solution
5	4	2:2	3:00	300	Light yellow solution
6	5	2.5:2.5	3:00	300	Light yellow solution
7	5	3.5:1.5	3:00	300	Light yellow solution
8	5	3:2	3:00	300	Colorless solution
**9**	**5**	**4:1**	**3:00**	**300**	**Clear and colorless solution**

SUPPLEMENTARY TABLE 2. Optimization of the digestion temperature for a 0.5 g sample.

№	Total volume	Volume ratio in ml HNO_3:$HClO_4$	Time	Temperature in ˚C	Observations
**1**	**5**	**4:1**	**3:00**	**300**	**Clear and colorless solution**
2	5	4:1	3:00	270	Colorless solution
3	5	4:1	3:00	240	Light Yellow solution
4	5	4:1	3:00	210	Light yellow solution
5	5	4:1	3:00	180	Yellow solution
6	5	4:1	3:00	140	Deep Yellow solution

SUPPLEMENTARY TABLE 3. Optimization of the digestion time

№	Total volume	Volume ratio in ml HNO3: HClO4	Time	Temperature (˚C)	Observation during the Volume ratio in Optimization
1	5	4:1	3:00	300	Clear and colorless solution
2	5	4.1	2:30	300	Colorless solution
3	5	4:1	2:00	300	Light yellow solution
4	5	4:1	1:30	300	Yellow solution
5	5	4:1	1:00	300	Deep yellow solution
